# Supplementary material for: Identification and Comprehensive Structural and Functional Analyses of the EXO70 Gene Family in Cotton
Source: Genes (Basel). 2021 Oct 9;12(10):1594. doi: 10.3390/genes12101594 (PMC8536163; doi:10.3390/genes12101594)
Supplement: Supplementary file 1 [file genes-12-01594-s001.zip › genes-1393683-supplementary.pdf]

Table S1. Primers used in this study.

| Primer name | Gene ID | Sequence（5'-3'） |
|---|---|---|
| GhEXO70A1-F | Gh_A10G1765 | ATGGGAATAGCAGTTGCAGG |
| GhEXO70A1-R | Gh_A10G1765 | CTATCGTTTTGGTTCATTTATATTCTT |
| VIGS-GhEXO70A1-F | Gh_A10G1765 | TGCCTGCAGACTAGTTCAATGATGAGGGAAGCTCTCC |
| VIGS-GhEXO70A1-R | Gh_A10G1765 | ACCTAGGGGCGCGCCAAAGCGAATGTTGCTTCTTAGCT |
| 2300-GhEXO70-F | Gh_A10G1765 | CGAACGATATCTAGAATGGGAATAGCAGTTGCAGG |
| 2300-GhEXO70-R | Gh_A10G1765 | CACCATGTTAATTAATCGTTTTGGTTCATTTATATTCTTG |
| Q-GhEXO70A1-F | Gh_A10G1765 | AAACCATTACGGACAACGTCGTCT |
| Q-GhEXO70A1-R | Gh_A10G1765 | CTTCCGCAGACTTCAAAGACTTGTCT |
| Q-GhUBQ7-F | DQ116441 | GAAGGCATTCCACCTGACCAAC |
| Q-GhUBQ7-R | DQ116441 | CTTGACCTTCTTCTTCTTGTGCTTG |
| PGBK-EXO70A1-F | Gh_A10G1765 | ATGGAGGCCGAATTCATGGGAATAGCAGTTGCAGG |
| PGBK-EXO70A1-R | Gh_A10G1765 | CAGGTCGACGGATCCCTATCGTTTTGGTTCATTTATATTCTT |
| PGAD-GhEXO84A-F | Gh_D02G1716.1 | ATGAGCATAGGTGATGTATCGG |
| PGAD-GhEXO84A-R | Gh_D02G1716.1 | CTACAGACCTTGGTTACTTCCTTGAG |
| PGAD-GhEXO84B-F | Gh_A10G0069.1 | ATGGCGACGGCGAAGGCG |
| PGAD-GhEXO84B-R | Gh_A10G0069.1 | CTAGTAACTCCCATGCGATCTAACAGATG |
| PGAD-GhEXO84C-F | Gh_A06G1976.1 | ATGATGGAGAGCAGTGAAGAAG |
| PGAD-GhEXO84C-R | Gh_A06G1976.1 | TTAGCTCTCTTGATCAGTAAAAGTAGG |
| PGAD-GhSEC5-F | Gh_A02G0663.1 | ATGTCAACCGACAGCGATG |
| PGAD-GhSEC5-R | Gh_A02G0663.1 | TTATCTGCGCCTACGCTGA |
| PGAD-GhSEC6-F | Gh_A10G0602.1 | ATGATGGTCGAGGATCTGGG |
| PGAD-GhSEC6-R | Gh_A10G0602.1 | TTAAGTGAGTTTACGCCATATATAACC |
| PGAD-GhSEC8-F | Gh_D04G0009.1 | ATGGGGATTTTTGATGGATTTC |
| PGAD-GhSEC8-R | Gh_D04G0009.1 | TTATCTAAATTCAGGTGCAACTTT |
| PGAD-GhSEC10-F | Gh_A12G2497.1 | ATGCCAGAGAGGTCAAAATCTTC |
| PGAD-GhSEC10-R | Gh_A12G2497.1 | TCAACTAGAACCCGACCATAAAG |
| PGAD-GhSEC15A-F | Gh_D13G1260.1 | ATGGATTCCAAACCCAAGAAG |
| PGAD-GhSEC15A-R | Gh_D13G1260.1 | TCAGTTGAAGTCTTTCAATCTTTT |
| PGAD-GhSEC15B-F | Gh_D11G1191.1 | ATGAAGTCAACGAGGCCACG |
| PGAD-GhSEC15B-R | Gh_D11G1191.1 | TTAGCTCACATCCCGGAGTC |

Table S2. Predicted EXO70 genes from Arabidopsis thaliana and the corresponding proteins.

| Gene Name | Gene ID | Gene Models | Exon Number | Protein Length (aa) |
|---|---|---|---|---|
| *AtEXO70A1-1* | AT5G03540 | AT5G03540.1 | 12 | 638 |
| *AtEXO70A1-2* | AT5G03540 | AT5G03540.2 | 11 | 523 |
| *AtEXO70A1-3* | AT5G03540 | AT5G03540.3 | 13 | 664 |
| *AtEXO70A2* | AT5G52340 | AT5G52340.1 | 11 | 631 |
| *AtEXO70A3* | AT5G52350 | AT5G52350.1 | 9 | 586 |
| *AtEXO70B1* | AT5G58430 | AT5G58430.1 | 1 | 624 |
| *AtEXO70B2* | AT1G07000 | AT1G07000.1 | 2 | 599 |
| *AtEXO70C1* | AT5G13150 | AT5G13150.1 | 1 | 653 |
| *AtEXO70C2* | AT5G13990 | AT5G13990.1 | 1 | 695 |
| *AtEXO70D1* | AT1G72470 | AT1G72470.1 | 1 | 633 |
| *AtEXO70D2* | AT1G54090 | AT1G54090.1 | 1 | 622 |
| *AtEXO70D3* | AT3G14090 | AT3G14090.1 | 1 | 623 |
| *AtEXO70E1* | AT3G29400 | AT3G29400.1 | 1 | 658 |
| *AtEXO70E2-1* | AT5G61010 | AT5G61010.1 | 1 | 639 |
| *AtEXO70E2-2* | AT5G61010 | AT5G61010.2 | 1 | 639 |
| *AtEXO70F1* | AT5G50380 | AT5G50380.1 | 1 | 683 |
| *AtEXO70G1* | AT4G31540 | AT4G31540.1 | 1 | 687 |
| *AtEXO70G2* | AT1G51640 | AT1G51640.1 | 1 | 660 |
| *AtEXO70H1* | AT3G55150 | AT3G55150.1 | 1 | 636 |
| *AtEXO70H2* | AT2G39380 | AT2G39380.1 | 1 | 637 |
| *AtEXO70H3* | AT3G09530 | AT3G09530.1 | 1 | 637 |
| *AtEXO70H4* | AT3G09520 | AT3G09520.1 | 1 | 628 |
| *AtEXO70H5* | AT2G28640 | AT2G28640.1 | 2 | 605 |
| *AtEXO70H6* | AT1G07725 | AT1G07725.1 | 2 | 615 |
| *AtEXO70H7-1* | AT5G59730 | AT5G59730.1 | 1 | 634 |
| *AtEXO70H7-2* | AT5G59730 | AT5G59730.2 | 1 | 632 |
| *AtEXO70H8* | AT2G28650 | AT2G28650.1 | 1 | 573 |

Table S3. Predicted EXO70 proteins and the corresponding gene from Oryza sativa.

| Gene name | Gene Locus | Exon Number | Protein Length (aa) |
|---|---|---|---|
| *OsEXO70A1* | Os04g0685600 | 12 | 634 |
| *OsEXO70A2* | Os11g0157400 | 12 | 643 |
| *OsEXO70A3* | Os12g0159700 | 18 | 976 |
| *OsEXO70A4* | Os04g0685500 | 12 | 661 |
| *OsEXO70B1* | Os01g0827500 | 1 | 652 |
| *OsEXO70B2* | Os05g0473500 | 1 | 661 |
| *OsEXO70B3* | Os01g0827600 | 4 | 553 |
| *OsEXO70C1* | Os12g0165600 | 1 | 700 |
| *OsEXO70C2* | Os11g0167600 | 1 | 692 |
| *OsEXO70D1* | Os08g0455700 | 1 | 632 |
| *OsEXO70D2* | Os09g0439600 | 1 | 638 |
| *OsEXO70E1* | Os01g0763700 | 1 | 602 |
| *OsEXO70F1* | Os02g0505400 | 1 | 689 |
| *OsEXO70F2* | Os04g0382200 | 1 | 688 |
| *OsEXO70F3* | Os01g0921400 | 3 | 556 |
| *OsEXO70F4* | Os08g0530300 | 1 | 606 |
| *OsEXO70F5* | Os10g33850 | 3 | 461 |
| *OsEXO70G1* | Os02g0149700 | 1 | 494 |
| *OsEXO70G2* | Os06g0698600 | 1 | 673 |
| *OsEXO70G3* | Os08g0519900 | 2 | 687 |
| *OsEXO70H1a* | Os11g0650100 | 1 | 579 |
| *OsEXO70H1b* | Os11g0649900 | 1 | 579 |
| *OsEXO70H2* | Os03g0448200 | 1 | 556 |
| *OsEXO70H3* | Os12g0100700 | 3 | 590 |
| *OsEXO70H4* | Os11g0100800 | 3 | 590 |
| *OsEXO70I1* | Os01g0905300 | 1 | 381 |
| *OsEXO70I2* | Os01g0905200 | 2 | 557 |
| *OsEXO70I3* | Os04g0111500 | 3 | 398 |
| *OsEXO70I4* | Os07g0210300 | 5 | 691 |
| *OsEXO70I5* | Os07g0210900 | 5 | 588 |
| *OsEXO70I6* | Os07g0210000 | 4 | 646 |
| *OsEXO70J1* | Os08g0232700 | 1 | 526 |
| *OsEXO70J2* | Os09g0347300 | 1 | 598 |
| *OsEXO70J3* | Os05g0369500 | 1 | 528 |
| *OsEXO70J5* | Os05g0369300 | 1 | 520 |
| *OsEXO70J6* | Os01g0383100 | 3 | 681 |
| *OsEXO70J7* | Os02g0575900 | 3 | 700 |
| *OsEXO70J8* | Os06g0183600 | 1 | 486 |
| *OsEXO70K1* | Os06g0255900 | 1 | 412 |
| *OsEXO70K2* | Os07g0211000 | 3 | 426 |
| *OsEXO70L1* | Os11g0572200 | 2 | 433 |

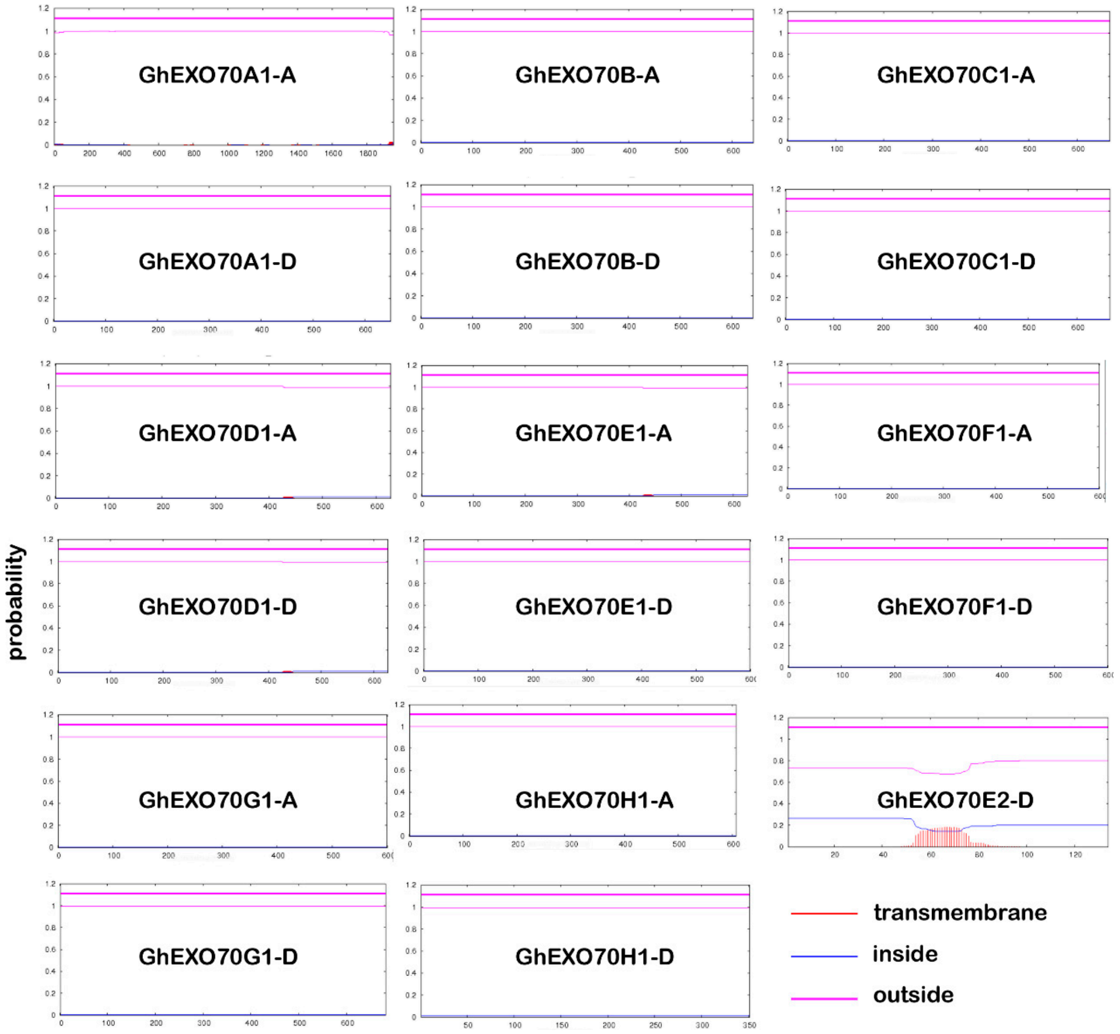


Figure S1. Analysis of transmembrane domains of EXO70 gene family in upland cotton.

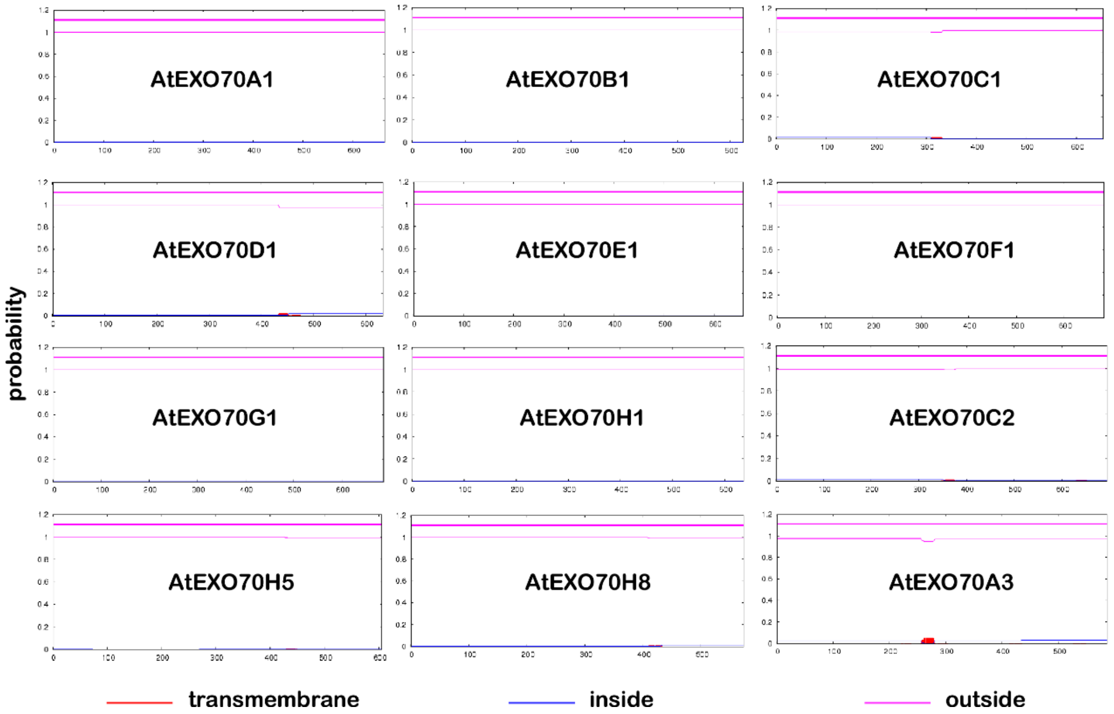


Figure S2. Analysis of transmembrane domains of EXO70 gene family in Arabidopsis thaliana.

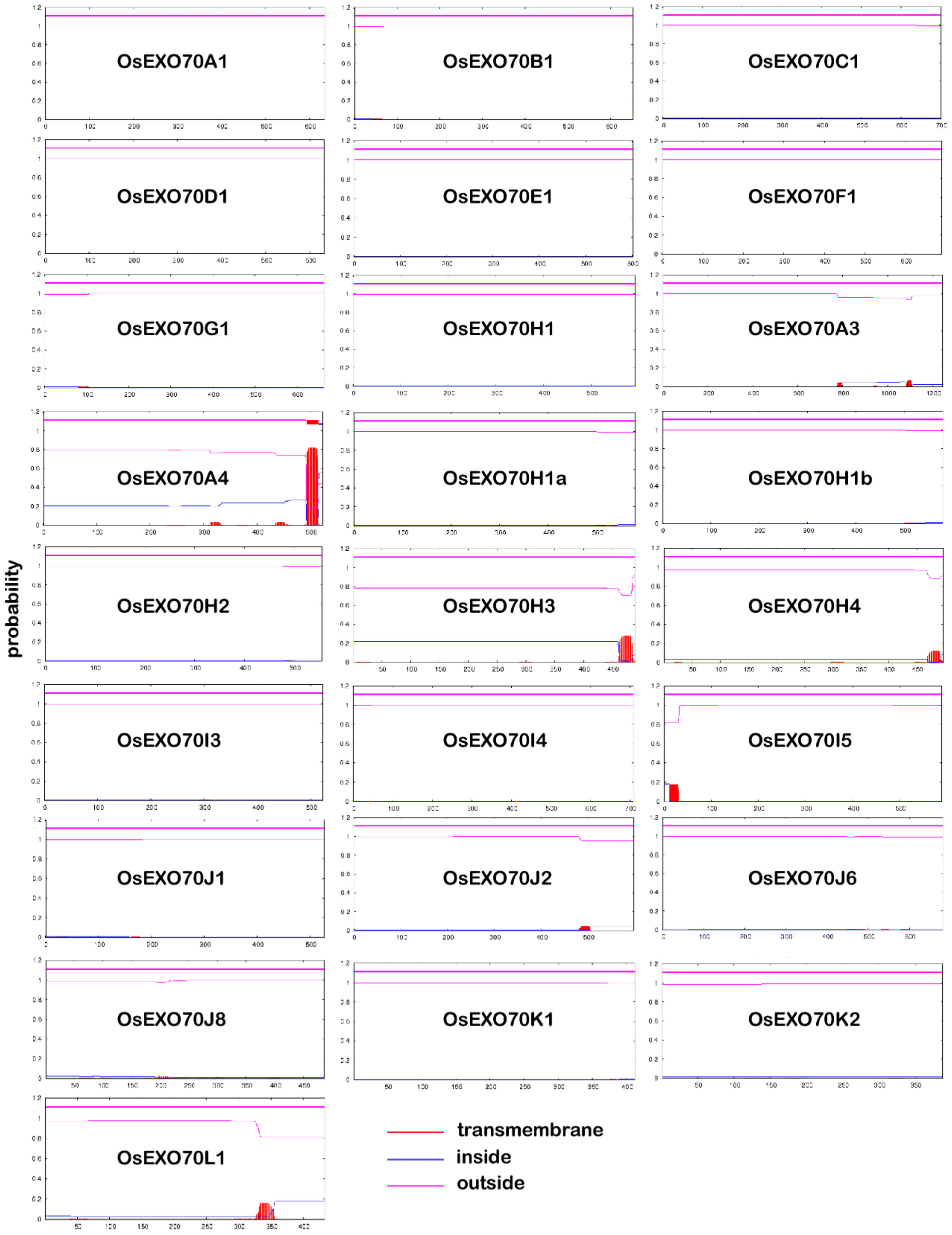


Figure S3. Analysis of transmembrane domains of EXO70 gene family in rice.

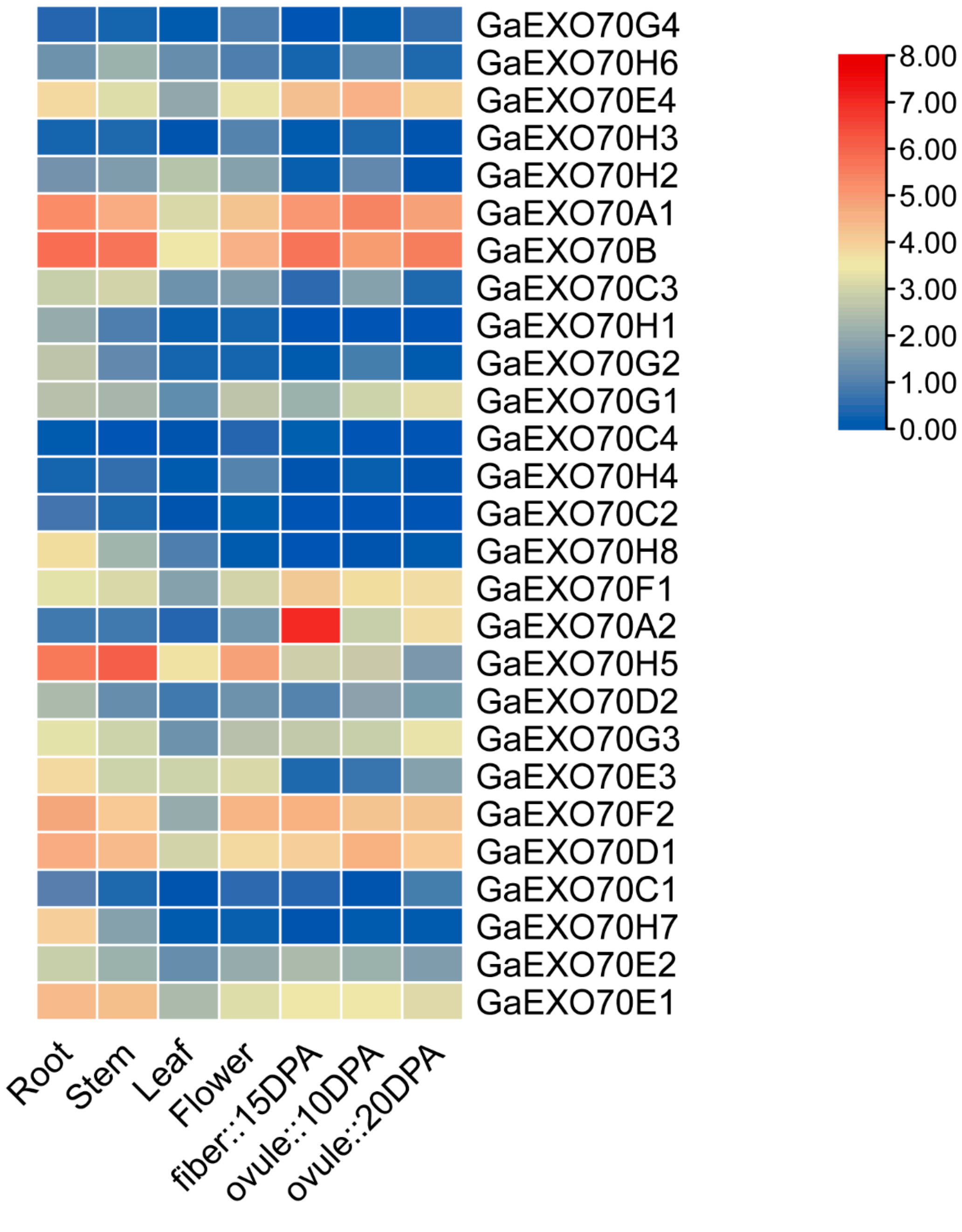


Figure S4. EXO70 family gene expression patterns in different tissues and organs of asian cotton.

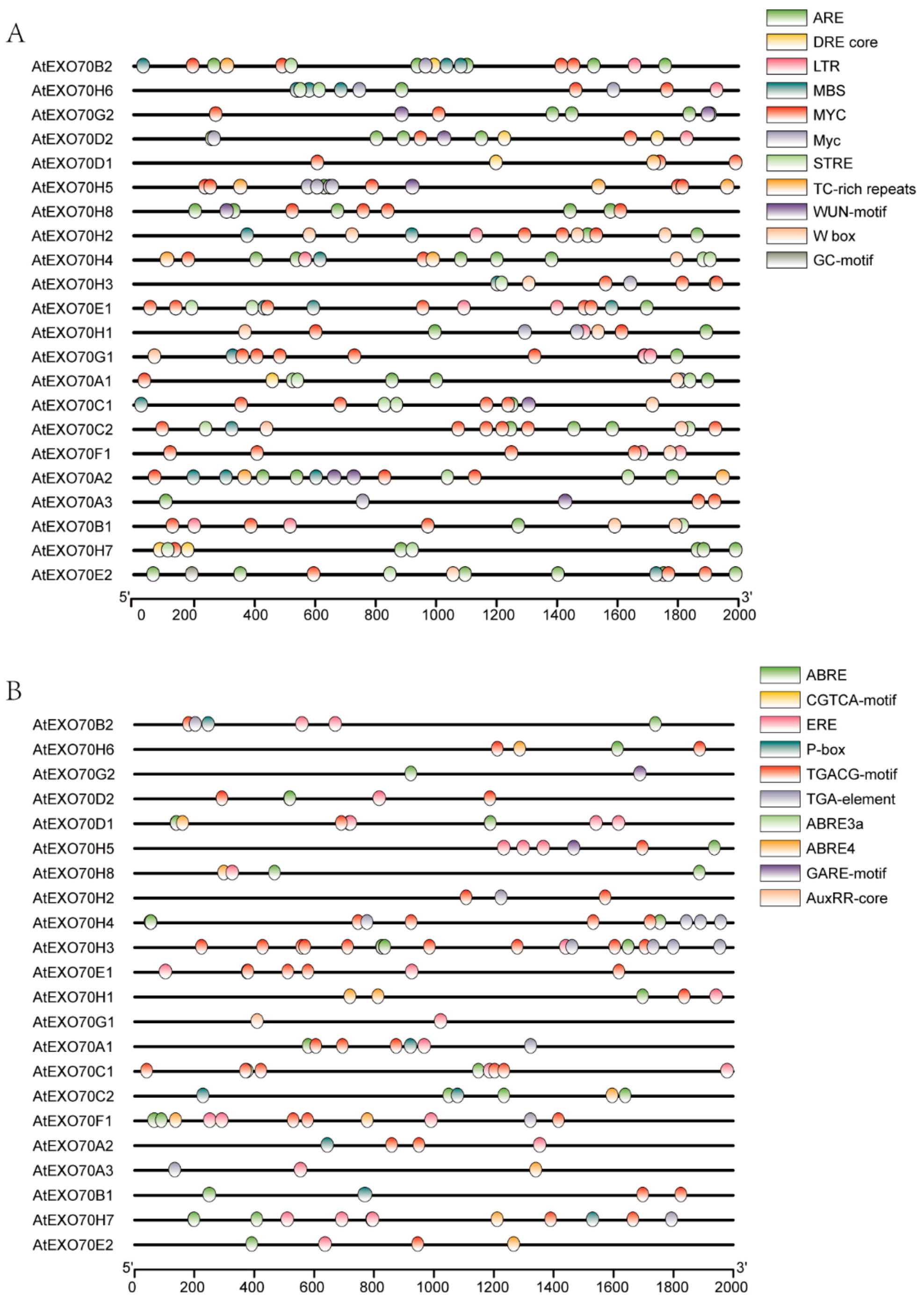


Figure S5. Cis-acting elements in Arabidopsis EXO70s promoter. (A) Predicted cis-elements involved in environmental stress response. ARE: cis-acting regulatory element for anaerobic induction. DRE core: dehydration response element. LTR: cis-acting elements involved in low temperature response. MBS: MYB binding sites related to drought induction. MYC: cis-acting elements involved in drought stress. STRE: stress response elements. TC-rich repetitive sequences: cis-acting elements involved in defense and stress responses. WUN-motif: wound response elements. W box: cis-acting elements involved in sugar metabolism and plant defense signals. GC-motif: enhancer-like elements involved in specific hypoxia induction. (B) Cis-elements involved in

phytohormones were predicted. ABRE: cis-acting regulatory element involved in abscisic acid response. CGTCA-motif: cis-acting regulatory element involved in methyl jasmonate (MeJA) response. ERE: cis-acting ethylene response element. P-box: gibberellin response element. TGACG-motif: cis-acting regulatory element involved in MeJA response. TGA-element: auxin response element. GARE-motif: gibberellin response element. AuxRR-core: cis-acting regulatory element involved in auxin response.

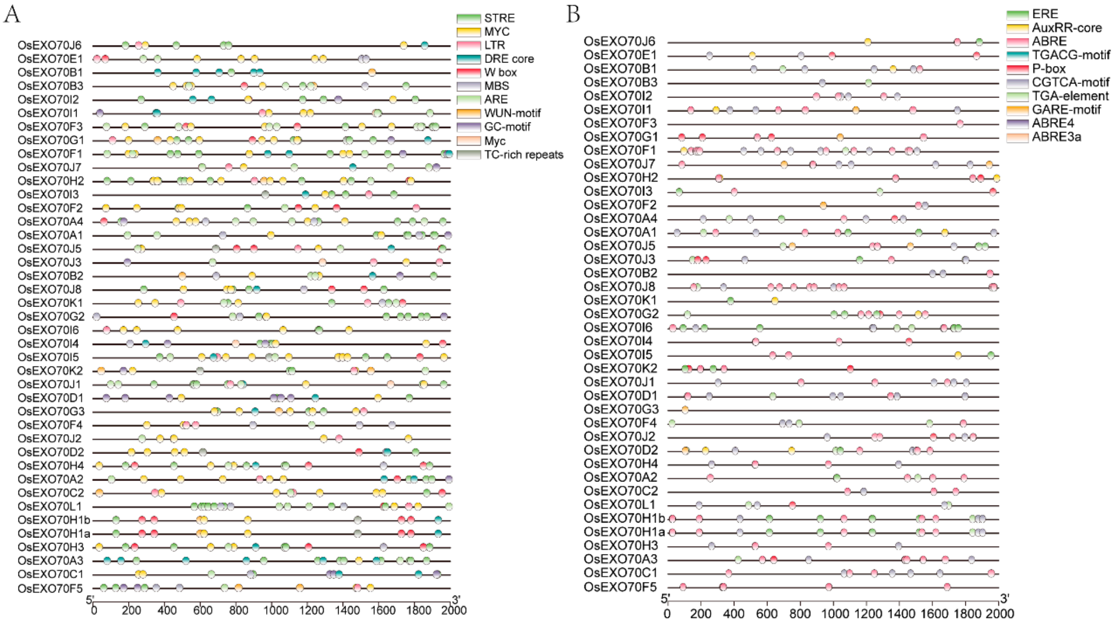


Figure S6. Cis-acting elements in rice EXO70s promoter. (A) Predicted cis-elements involved in environmental stress response. STRE: stress response elements. MYC: cis-acting elements involved in drought stress. LTR: cis-acting elements involved in low temperature response. DRE core: dehydration response element. W box: cis-acting elements involved in sugar metabolism and plant defense signals. MBS: MYB binding sites related to drought induction. ARE: cis-acting regulatory element for anaerobic induction. WUN-motif: wound response elements. GC-motif: enhancer-like elements involved in specific hypoxia induction. TC-rich repetitive sequences: cis-acting elements involved in defense and stress responses. (B) Cis-elements involved in phytohormones were predicted. ERE: cis-acting ethylene response element. AuxRR-core: cis-acting regulatory element involved in auxin response. ABRE: cis-acting regulatory element involved in abscisic acid response. TGACG-motif: cis-acting regulatory element involved in MeJA response. P-box: gibberellin response element. CGTCA-motif: cis-acting regulatory element involved in methyl jasmonate (MeJA) response. TGA-element: auxin response element. GARE-motif: gibberellin response element.

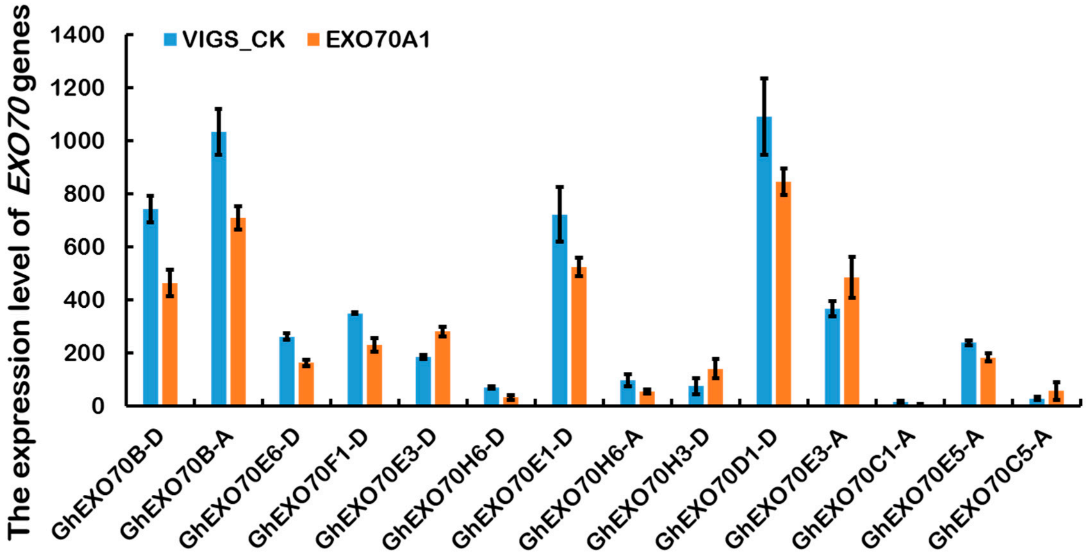


Figure S7. Expression analysis of other changed EXO70 genes in NGS data.
